# Association of serum fatty acid pattern with depression in U.S. adults: analysis of NHANES 2011–2012

**DOI:** 10.1186/s12944-024-02142-9

**Published:** 2024-06-08

**Authors:** Hengying Chen, Jue Wang, Baohua Zheng, Wenqi Xia, Gongjun Tan, Hongyuan Wu, Yao Wang, Zhen Deng, Yiyuan Wang, Jianduan Zhang, Hongzhong Zhang

**Affiliations:** 1 Department of Maternal and Child Health, School of Public Health, Tongji Medical College, Huazhong University of Science and Technology, Wuhan, Hubei China; 2 Huadu District Center for Disease Control and Prevention, Guangzhou, Guangdong China; 3 Department of Clinical Laboratory, Zhuhai Center for Maternal and Child Health Care, Zhuhai, Guangdong China; 4 Department of Child Health, Zhuhai Center for Maternal and Child Health Care, Zhuhai, Guangdong China; 5 Postnatal Care Center (Department of Postpartum Rehabilitation), Zhuhai Center for Maternal and Child Health Care, Zhuhai, Guangdong China; 6 Key Laboratory of Environment and Health, Ministry of Education & Ministry of Environmental Protection, State Key Laboratory of Environmental Health (Incubation), School of Public Health, Tongji Medical College, Huazhong University of Science and Technology, Wuhan, Hubei China; 7 Zhuhai Center for Maternal and Child Health Care, Zhuhai, Guangdong China

**Keywords:** Fatty acids, Depression, Pattern analysis, National Health and Nutrition Examination Survey

## Abstract

**Background:**

Exposure to different concentration levels of fatty acids (FAs) may have an impact on depression. However, previous studies using individual FAs may not reflect the performance of mixtures of various FAs, and the associations of FA patterns with depression remain unclear.

**Methods:**

We conducted the cross-sectional analysis in 792 adults aged 18 and older with available serum FAs and depression screening data in the National Health and Nutrition Examination Survey (NHANES) 2011–2012. The serum concentrations of thirty FAs were measured using gas chromatography-mass spectrometry and their percentage compositions were subsequently calculated. Depression was defined as the Patient Health Questionnaire-9 score ≥ 10. We employed principal component analysis to derive serum FA patterns. We examined the association between these patterns and depression in the overall population and various subgroups through survey-weighted logistic regression.

**Results:**

Four distinct patterns of serum FAs were identified: ‘high eicosapentaenoic acid (EPA) and docosahexaenoic acid (DHA); low docosatetraenoic acid (DTA) and docosapentaenoic acid (DPA) n-6’, ‘high long-chain saturated FA and long chain FA’, ‘low median-chain saturated FA and myristoleic acid’ and ‘low capric acid and lauric acid; high gamma-linolenic acid (GLA) and stearidonic acid (SDA)’ pattern. Individuals in the high tertile of ‘high EPA and DHA; low DTA and DPA n-6’ pattern score had 0.46 (95% CI: 0.22, 0.93) lower odds of developing depression compared to individuals in the lowest tertile after adjusting for confounders such as age, sex, physical activity and total energy intake, etc. The odds ratio (OR) of depression was increased in the population with the highest tertile of ‘low capric acid and lauric acid; high GLA and SDA’ pattern (OR: 2.45, 95% CI: 1.24, 4.83). In subgroup analyses, we observed that the association between ‘high EPA and DHA; low DTA and DPA n-6’ and depression persisted among specific demographic and lifestyle subgroups, including females, non-Mexican Americans, non-obese, those aged over 60 years, smokers and drinkers. Similarly, ‘low capric acid and lauric acid; high GLA and SDA’ showed stable associations in female, non-Mexican Americans and smokers.

**Conclusions:**

Serum FA patterns are associated with depression, and their relationships vary across sex, race, BMI, age, smoking and drinking subgroups, highlighting the importance of considering specific FA patterns within these demographic and lifestyle categories. Utilization of combined FA administration may serve as a mitigation measure against depression in these specific populations.

**Supplementary Information:**

The online version contains supplementary material available at 10.1186/s12944-024-02142-9.

## Introduction

Depression, a common mental disorder characterized not only by persistent feelings of sadness and anhedonia but also by cardinal symptoms such as sleep disturbances, changes in appetite, decreased energy, feelings of guilt and suicidal ideation, constitutes a major public health problem [1, 2]. Globally, approximately 280 million individuals were affected by depressive disorders, contributing to more than 47 million disability-adjusted life-years in 2019 [3]. Alarmingly, the projections indicate that depression will become the leading cause of disease burden by 2030 [2]. Unfortunately, current treatment options remain suboptimal for some affected individuals, as not all patients respond favorably to available antidepressant medications, and some may experience intolerable side effects [4, 5]. Diets such as the Mediterranean pattern, due to their non-toxic nature and daily consumption, have been studied and suggested to play a potentially protective role against depression, which highlights the importance of consuming fish [6, 7]. However, the mechanisms triggered by these dietary patterns remain poorly understood.

Fish are highly enriched in fatty acids (FAs). Research into the therapeutic effects and mechanisms of polyunsaturated fatty acids (PUFA) or other types of FAs in depression has gained intensive attention since the discovery of the inverse association between fish consumption and depression prevalence in 1998 [8]. FAs play a vital role in maintaining the composition and fluidity of neuronal membranes, regulating neurotransmitter systems and gene expression, and impacting the neuroinflammatory processes. Therefore, FAs have been considered a potential favorable intervention for alleviating depression [9, 10]. Mounting randomized controlled trials (RCTs) have examined the efficacy of omega-3 (n-3) PUFA in treating depression [11, 12]. However, previous systematic reviews and meta-analyses have shown diverse outcomes of n-3 PUFA supplementation on depressive symptoms [12–14]. Concerns have been voiced regarding the quality of evidence from these RCTs and the potential for publication bias [15]. Emphasis has been placed on the importance of using EPA-rich formulations and examining clinically diagnosed depressed populations in PUFA supplementation studies [16, 17]. As for other FAs, several epidemiological studies have indicated the notable impacts of circulating specific omega-6 (n-6) PUFAs, monounsaturated fatty acids (MUFA) and saturated fatty acids (SFA) on anxiety and depression [18, 19]. However, other epidemiological analyses failed to establish a significant correlation between depressive symptoms and SFA, MUFA, and n-6 PUFA levels [20–22], leaving the associations between different subtypes of FAs and depression inconclusive.

Previous studies primarily focused on specific individual FAs (e.g., docosahexaenoic acid [DHA] and eicosapentaenoic acid [EPA]) or groups of FAs (e.g., SFA, MUFA, and PUFA). However, in real-life scenarios, these components often co-exist, and an exclusive focus on individual FAs or groups could have overlooked their potential synergistic or additive effects on depression, oversimplifying the complex interplays among FAs composition [23]. Moreover, some FAs interact through different pathways, such as elongation and desaturation. For instance, alpha-linolenic acid (ALA) is formatted to stearidonic acid (SDA) through delta-6 desaturase. In contrast, docosahexaenoic acid (DHA) is synthesized from docosapentaenoic acid (DPA) by further chain elongation, D6-desaturation and limited peroxisomal oxidation [24]. Therefore, studies investigating the impact of FAs on depression should account for their complex interactions with methods such as principal component analysis (PCA) that incorporate correlations to address these concerns. While some studies have applied this method to identify novel patterns of circulating FAs and have documented associations between these patterns and various diseases, including prostate cancer, allergy and cardiometabolic health [25–27], the relationship between patterns of circulating FAs and depression remains unclear.

This study aims to bridge the aforementioned gaps by investigating the associations between serum FA patterns and depression in U.S. adults, using data from the National Health and Nutrition Examination Survey (NHANES) database.

## Methods

### Study population

NHANES, an ongoing initiative under the National Center for Health Statistics (NCHS) within the Centers for Disease Control and Prevention (CDC), employs a meticulously structured, multistage probability design to collect data on health and nutrition. This data is compiled from a nationally representative sample of the community-dwelling population in the United States [28]. The study design and procedure details, including demographics, questionnaires, socioeconomic status and laboratory results, are readily accessible to the public via the following online resource (https://www.cdc.gov/nchs/nhanes/). The NCHS ethics review board approved the NHANES protocols and written informed consent was obtained from all participants [28].

For this study, we used data from NHANES 2011–2012 (*n* = 9756), as it provided information on both serum FA concentrations and depression during this particular survey cycle [29]. Further, we limited our participants to those aged 18 or older with available serum FA data and completed the depression screening questionnaires. Data from NHANES 2011–2012 “Fatty Acids - Serum (FAS_G)” was used in conjunction with “Mental Health-Depression Screener (DPQ_I)” to analyze a potential relationship between serum FA patterns and depression [30]. Demographic data was sourced from the “Demographic Variables and Sample Weights (DEMO_I)” Report [31]. The depression screening was given at the mobile examination center to participants 12 years and older; however, only responses from participants 18 and older were included in the NHANES data file. Therefore, the final sample size consisted of 792 adults.

### Outcomes

The Patient Health Questionnaire (PHQ-9), a nine-item screening instrument that assesses the frequency of various depressive symptoms within the past two weeks, was used to screen for depression in NHANES [32]. Each item was scored from 0 (“not at all”) to 3 (“nearly every day”), with the sum of all scores representing the overall severity of the depression on a scale of 0–27. Higher scores indicate higher levels of depressive symptoms and scores are categorized as follows: mild (5–9), moderate (10–14), moderately severe (15–19) and severe levels of depressive symptoms (20–27). A cut point for inclusion into the depression group was a score of ≥ 10, which achieved 88% sensitivity and 88% specificity for detecting clinical depression [33].

### Exposures

Thirty subtypes of FAs, including eleven SFA, six MUFA, and thirteen PUFA, were measured in fasting serum samples using gas chromatography/mass spectrometry at the Division of Laboratory Sciences, U.S. CDC (Table S1) [33]. FAs were measured using modified Lagerstedt methods described previously [34]. Briefly, total FAs were extracted with hexane, along with an internal standard solution, to ensure accurate FA recovery. The resulting extract was then derivatized to form pentafluorobenzyl esters and injected into a capillary gas chromatograph column. FAs were expressed as a % of total FAs (FAs/the sum of measured FAs). Values of FAs below the limit of detection (LOD) were replaced by LOD divided by the square root of 2 [30].

**Fig. 1:**
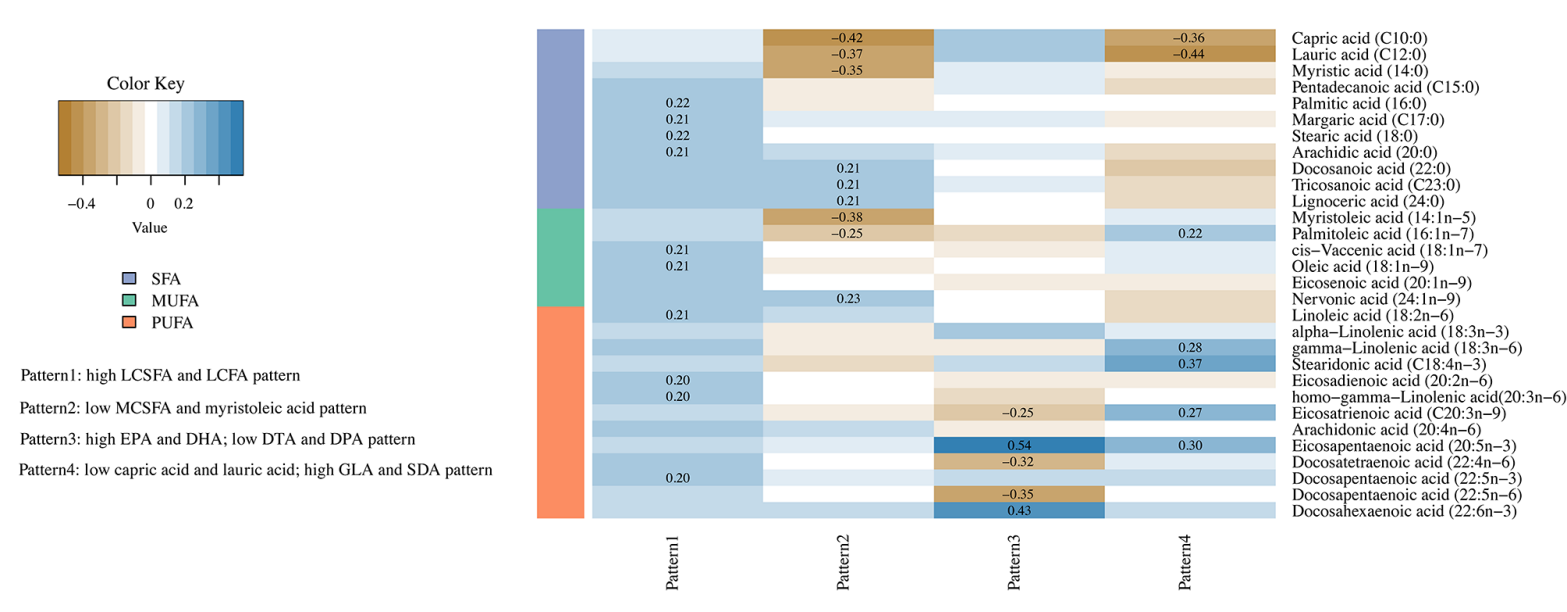
Factor loading matrix of fatty acids for the major serum fatty acids pattern. LCSFA: long-chain saturated fatty acids, MCSFA: medium-chain saturated fatty acids, LCFA: long-chain fatty acids, EPA: eicosapentaenoic acid, DHA: docosahexaenoic acid, DPA: docosapentaenoic acid, DTA: docosatetraenoic acid, GLA: gamma-linolenic acid, SDA: stearidonic acid

PCA with varimax rotation was used to identify FA patterns. The rationality of the data structure was confirmed through the Kaiser–Meyer–Olkin test (KMO = 0.68) and the Bartlett test of sphericity (*P* < 0.01). The number of patterns was selected considering a scree plot combined with the eigenvalue (> 1) and factor interpretability. Our study’s first four components with eigenvalues > 1 explained 63.1%, 9.8%, 5.6%, and 4.5% of the total variance, respectively. Additionally, the scree plot exhibited a gentle decline beyond the fourth principal component (Fig. S1). Thus, four FA patterns were determined. FA patterns were labeled according to the FAs with high factor loadings (≥|0.20|) for each pattern (Fig. 1). Each participant was assigned an individual score for the derived FA patterns, calculated as serum FA levels multiplied by the respective FA factor loading, with summation across all thirty FAs. A score for a specific FA pattern represents a weighted sum of all thirty FAs, and a higher score indicates greater conformity with the identified FA pattern.

### Covariates

The selection of covariates is based on previous literature and a directed acyclic graph (DAG). Sociodemographic factors including sex, age (as a continuous variable), race/ethnicity (Mexican-American, non-Hispanic white, non-Hispanic black, other/multiracial), smoking (ever smoker or never smoker) and drinking (ever drinker or never drinker), body mass index (BMI, as a continuous variable), total energy intake (as a continuous variable) and physical activity (not reported, < 500 metabolic equivalents [MET]-min/week, 500–1000 MET-min/week, > 1000 MET-min/week) were examined as potential confounders based on the minimal sufficient adjustment set suggested by DAGitty (Fig. S2) [35]. According to prior literature, education (less than 9th grade or 9-11th grade, high school graduate, some college or associate degree, college graduate or above), annual family income (Under $20,000, $20,000 and over), marital status (married or living with a partner, widowed/divorced/separated, never married) were also added for adjustment as related variables [36–41]. Individuals who had never smoked or had smoked less than 100 cigarettes in their lifetime were defined as never smokers; otherwise, as ever smokers. Participants who reported consuming less than twelve alcoholic drinks yearly were labeled never drinkers, while those consuming at least twelve annually were categorized as ever drinkers.

### Statistical analysis

All analyses were weighted using survey design procedures, accounting for the effects of sampling design stratification and clustering procedures. According to the recommendations of NHANES, sampling weights associated with the smallest subsample were used for analyses [42].

Baseline characteristics of participants were expressed as frequency (weighted percentages) for categorical variables and mean ± standard deviation for continuous variables by depression status. A Chi-square test was applied to compare the distribution of categorical variables such as sex, education, and race/ethnicity by depression status. The t-tests were used to compare the mean levels of continuous variables between depressive participants and those without depression. One-way analysis of covariance (ANCOVA) adjusted for confounders were used to compare the mean difference between factor scores among the severity of depression. The *P*-diff value from ANCOVA tested group differences by including depression severity as a categorical variable and examined its relationship with FA pattern scores. Additionally, the *P* value for trend from ANCOVA assessed the linear relationship between increasing depression severity and FA pattern scores by modeling depression severity as an ordinal variable (none = 0, mild depressive symptoms = 1, moderate depressive symptoms = 2, moderately severe depressive symptoms = 3, severe depressive symptoms = 4). Weighted logistic regression was used to calculate odds ratios (ORs) and 95% confidential intervals (CIs) for depression for each tertile of factor scores with the lowest tertile (T1) as the reference, and four different logistic regression models were performed. Model 1 was the unadjusted model, and Model 2 was adjusted for age and sex. Model 3 included the covariates of Model 2 with additional adjustments for race/ethnicity, education, marital status, annual family income, BMI, alcohol status, and smoking status. Model 4 included the covariates of Model 3 with additional adjustments for physical activity and total energy intake. A *P* value for the trend was calculated by including the tertiles as a continuous variable in the logistic regression model.

Two sensitivity analyses were performed: (1) using the PHQ-9 score as a continuous variable to assess the association of depression with factor scores by linear regression to assess the robustness of our results; (2) to address the potential differences in results arising from variations in concentrations and percent composition, additional analyses were conducted using FA concentration as the primary exposure variable. All analyses were conducted using R version 4.3.1 (R Foundation, Vienna, Australia). All tests were two-sided, and alpha was set at *P* < 0.05, presenting a significance level unless otherwise stated.

## Results

### Population characteristic

The study sample comprised 792 participants, of whom about half were female (53.28%). Nearly 15% of the sample (15.40%) had depression. The demographics of the study population by depression status from NHANES (2011–2012) are shown in Table 1. Participants with depression were more likely to have a higher BMI (*P* < 0.01), a lower annual family income (*P* < 0.01) and a higher likelihood of being widowed, divorced, or separated (*P* < 0.01) compared with those without depressive symptoms. There were no significant differences in the other baseline characteristics between the two groups. In addition, the characteristics of participants for tertiles of each pattern score are shown in Table S2.

**Table 1:** Baseline characteristics of study participants by depression status

Characteristics	Depression (*n* = 122)	Non-depression (*n* = 670)	*P*
Age (years), Mean ± SD^a^	45.5 ± 14.94	46.1 ± 17.76	0.76
BMI (kg/m^2^), Mean ± SD^a^	30.9 ± 6.90	28.9 ± 6.73	0.01
Total energy (kcal), Mean ± SD^a^	1942.92 ± 938.12	2151.83 ± 789.28	0.07
Sex, n (%)^b^			0.10
Male	48 (39.2)	322 (49.9)	
Female	74 (60.8)	348 (50.1)	
Physical activity, n (%)^b^			0.06
Not reported	72 (44.5)	328 (55.3)	
<500 Met-min/week	19 (15.5)	94 (19.2)	
500–1000 Met-min/week	11 (12.4)	84 (11.8)	
>1000 Met-min/week	20 (27.7)	164 (13.6)	
Race/ethnicity, n (%)^b^			0.53
Mexican American	28 (14.7)	142 (13.7)	
Non-Hispanic whites	56 (72.4)	275 (70.7)	
Non-Hispanic black	32 (10.2)	158 (9.4)	
Other	6 (2.7)	95 (6.1)	
Education, n (%)^b^			0.74
Less than 9th grade or 9–11th grade	37 (20.2)	128 (15.5)	
High school graduate	29 (22.1)	136 (19.6)	
Some college or AA degree	31 (34.3)	206 (33.4)	
College graduate or above	19 (23.5)	171 (31.6)	
Annual family income, n (%)^b^			< 0.01
Under $20,000	58 (36.7)	173 (19.4)	
$20,000 and over	57 (63.4)	471 (80.6)	
Marital status, n (%)^b^			< 0.01
Married or living with partner	40 (37.7)	357 (61.1)	
Widowed/divorced/separated	53 (37.9)	138 (17.6)	
Never married	23 (24.5)	146 (21.3)	
Smoking, n (%)^b^			0.23
Yes	58 (49.3)	278 (43.4)	
No	58 (50.7)	362 (56.7)	
Drinking alcohol, n (%)^b^			0.10
Yes	81 (77.2)	504 (83.4)	
No	40 (22.8)	166 (16.6)	

MET: metabolic equivalent, AA: associates

^a^: *P* value was obtained from two-sample t test

^b^: unweighted sample size (weighted percentage); *P* value was obtained from the Chi-square test or Fisher’s exact when appropriate

### FA pattern across depression categories

FA pattern scores were compared according to different PHQ-9 categories in Table 2. The ANCOVA analysis showed that the ‘high EPA and DHA; low DTA and DPA n-6’ pattern score was significantly and inversely associated with the PHQ-9 score (*P*-diff < 0.01, *P*-trend = 0.02), while other pattern scores did not reach statistical significance.

**Table 2:** Comparisons of pattern score according to the severity of depression^a^

Variables	None (PHQ ≤ 4)	Mild depressive symptoms (5 ≤ PHQ ≤ 9)	Moderate depressive symptoms (10 ≤ PHQ ≤ 14)	Moderately severe depressive symptoms (15 ≤ PHQ ≤ 19)	Severe depressive symptoms (PHQ ≥ 20)	*P*-diff^b^	*P*-trend^c^
‘high LCSFA and LCFA’ pattern	1.81 ± 3.88	1.67 ± 4.15	1.84 ± 4.27	2.41 ± 3.73	-1.16 ± 3.88	0.70	0.42
‘low MCSFA and myristoleic acid’ pattern	0.02 ± 1.31	0.07 ± 1.17	-0.16 ± 1.49	-0.21 ± 1.52	-0.19 ± 0.90	0.21	0.73
‘high EPA and DHA; low DTA and DPA n-6’ pattern	0.30 ± 1.33	-0.36 ± 1.86	-0.17 ± 1.72	-1.33 ± 3.79	-0.52 ± 1.33	< 0.01	0.02
‘low capric acid and lauric acid; high GLA and SDA’ pattern	-0.01 ± 1.31	-0.09 ± 1.17	0.12 ± 1.49	-0.17 ± 1.52	0.55 ± 0.90	0.45	0.97

^a^: adjusted for sex, age, race/ethnicity, education, marital status, annual family income, body mass index, alcohol status, smoking status, physical activity and total energy intake

^b^: *P*-diff was obtained from ANCOVA using depression severity as a categorical variable

^c^: *P*-trend was obtained from ANCOVA using depression severity as an ordinal variable

### Association between FA patterns and depression

Table 3 presents the associations between FA pattern scores and the OR of depression. Compared with those without depression, the ORs and 95% CIs of depression for the highest tertile vs the lowest one of ‘high EPA and DHA; low DTA and DPA n-6’ pattern score were 0.41 (0.22, 0.77) in the crude analysis, 0.37 (0.18, 0.75) in Model 2, 0.44 (0.21, 0.91) in Model 3 and 0.46 (0.22, 0.93) in Model 4. The ‘low capric acid and lauric acid; high gamma-linolenic acid (GLA) and SDA’ pattern score was positively correlated with depression in the non-adjusted model (OR = 1.87, 95% CI = 1.06, 3.30), partially adjusted models (OR = 2.29, 95% CI = 1.27, 4.11; OR = 2.36, 95% CI = 1.19, 4.67) and fully adjusted model (OR = 2.45, 95% CI = 1.24, 4.83). In reference to the lowest tertile, participants in the second tertile of the ‘high LCSFA and LCFA’ pattern score had a significantly higher OR of depression, with an OR of 2.08 (95%CI: 1.01, 4.31) in Model 3. However, the significant association was attenuated to nonsignificance after additionally adjusting for total energy intake and physical activity (*P* = 0.15). In addition, there was no evidence of an association between tertiles of ‘low MCSFA and myristoleic acid’ pattern score and the odds of depression in crude and adjusted models (all *P* > 0.05).

**Table 3:** Odds ratios and 95% confidential intervals for depression (PHQ > 9) according to the tertiles of serum fatty acid pattern score

Variables	Tertile 1	Tertile 2	Tertile 3	*P*-trend
‘high LCSFA and LCFA’ pattern				
Cases/controls	33/230	53/211	36/228	
Model 1	Ref.	1.93 (1.03, 3.63)^*^	1.13 (0.56, 2.28)	0.53
Model 2	Ref.	1.99 (1.05, 3.75)^*^	1.16 (0.58, 2.30)	0.56
Model 3	Ref.	2.08 (1.01, 4.31)^*^	1.45 (0.65, 3.23)	0.96
Model 4	Ref.	2.05 (0.94, 4.48)	1.57 (0.64, 3.88)	0.73
‘low MCSFA and myristoleic acid’ pattern				
Cases/controls	48/215	41/223	32/232	
Model 1	Ref.	0.77 (0.33, 1.80)	0.62 (0.33, 1.15)	0.17
Model 2	Ref.	0.75 (0.32, 1.75)	0.56 (0.28, 1.14)	0.14
Model 3	Ref.	0.75 (0.29, 1.92)	0.60 (0.28, 1.27)	0.25
Model 4	Ref.	0.73 (0.29, 1.89)	0.62 (0.29, 1.35)	0.30
‘high EPA and DHA; low DTA and DPA n-6’ pattern				
Cases/controls	52/211	39/226	31/232	
Model 1	Ref.	0.73 (0.39, 1.36)	0.41 (0.22, 0.77)^*^	< 0.01
Model 2	Ref.	0.70 (0.39, 1.27)	0.37 (0.18, 0.75)^*^	< 0.01
Model 3	Ref.	0.63 (0.29, 1.37)	0.44 (0.21, 0.91)^*^	0.13
Model 4	Ref.	0.63 (0.29, 1.37)	0.46 (0.22, 0.93)^*^	0.40
‘low capric acid and lauric acid; high GLA and SDA’ pattern				
Cases/controls	27/236	46/219	48/215	
Model 1	Ref.	3.15 (1.67, 5.96) ^*^	1.87 (1.06, 3.30) ^*^	0.03
Model 2	Ref.	3.53 (1.75, 7.12) ^*^	2.29 (1.27, 4.11) ^*^	< 0.01
Model 3	Ref.	3.74 (1.83, 7.63) ^*^	2.36 (1.19, 4.67) ^*^	0.04
Model 4	Ref.	3.81 (1.89, 7.69) ^*^	2.45 (1.24, 4.83) ^*^	0.08

Ref: reference. ^*^: *P* < 0.05

Model 1: crude model

Model 2: adjusted for age and sex

Model 3: adjusted for model 2 plus race/ethnicity, education, marital status, annual family income, body mass index, alcohol status, and smoking status

Model 4: adjusted for model 3 plus physical activity and total energy intake

*P*-trend was obtained using the tertiles as a continuous variable

### Subgroup analyses

**Fig. 2:**
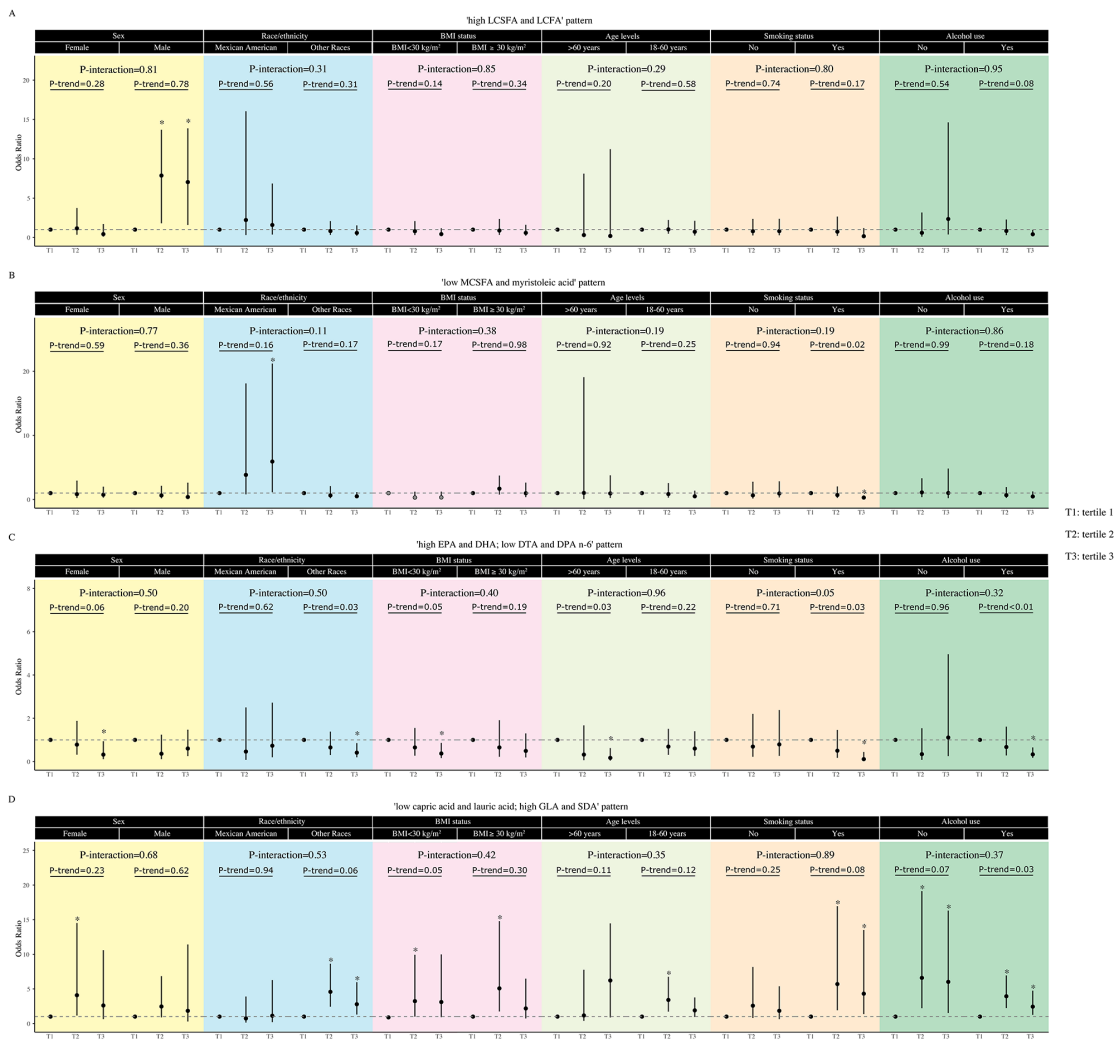
Odds ratios (95% CI) from multiple logistic regression analysis models of associations between serum fatty acid pattern score and odds ratios of depression in different subgroups. The subgroup analyses were adjusted for the covariates as in Model 4, excluding the stratification variable

The relevance between serum FA pattern score in tertile levels and depression in different subgroups is shown in Fig. 2. Males exhibited an escalating OR of depression within the population characterized by the T3 of the ‘high LCSFA and LCFA’ pattern score (OR = 7.04, 95% CI = 1.59, 13.87). Conversely, Mexican-Americans demonstrated an increasing OR of depression in the population associated with the T3 of the ‘low MCSFA and myristoleic acid’ pattern score (OR = 5.92, 95% CI = 1.12, 21.22). In addition, the significant effects of the ‘high EPA and DHA; low DTA and DPA n-6’ pattern on depression only remained in females, non-Mexican-American, non-obese, the elder group, smokers and alcohol users (all *P* < 0.05), and the significant interaction was detected for smoking status (*P*-interaction = 0.05). The subgroup analyses of the ‘low capric acid and lauric acid; high GLA and SDA’ pattern stratified by sex, race and smoking status was similar to that of the ‘high EPA and DHA; low DTA and DPA n-6’ pattern. Differently, younger and middle-aged had a significantly higher OR for depression in the population of T2 and the modification of BMI status and alcohol use was not observed.

### Sensitivity analyses

No appreciable attenuation was observed from the effects of the ‘high EPA and DHA; low DTA and DPA n-6’ pattern on depression when treating the PHQ-9 score as a continuous variable. However, the association with ‘low capric acid and lauric acid; high GLA and SDA’ pattern became nonsignificant (Table S3). When using FA concentration as the primary exposure instead of FA composition, we found that two FA grouping patterns remained consistent: the “LCSFA and LCFA” pattern and the “low MCSFA and myristoleic acid” pattern. Interestingly, a contrast emerged with the “low EPA and DHA; high DTA and DPA” pattern against the “high EPA and DHA; low DTA and DPA” pattern. The detrimental effect of the “low EPA and DHA; high DTA and DPA” pattern on depression was observed, confirming our findings. However, the grouping of the “high Capric acid and lauric acid” pattern, differing from the “low capric acid and lauric acid; high GLA and SDA” group, showed no notable effects on depression (Tables S4 and Fig. S3-S4).

## Discussion

Based on a comprehensive analysis of a nationally representative sample, we discerned four distinct serum FA patterns, including the ‘high LCSFA and LCFA’ pattern, ‘low MCSFA and myristoleic acid’ pattern, ‘high EPA and DHA; low DTA and DPA n-6’ pattern and ‘low capric acid and lauric acid; high GLA and SDA’ pattern in U.S. adults. The OR of depression was found to alter correspondingly with increasing scores for patterns characterized by ‘high EPA and DHA; low DTA and DPA n-6’ and ‘low capric acid and lauric acid; high GLA and SDA’. These significant associations primarily differed across sex, race, age, BMI, smoking status and alcohol use subgroups.

The PCA method revealed the four distinct FA patterns in serum samples. The complex FA synthesis pathways in the human body may contribute to the formation of diverse FA patterns [43]. For example, DTA can be converted into DPA with the catalyzation of Δ6-desaturase, which can then be elongated to DHA, offering a possible explanation for the identification of the ‘high EPA and DHA; low DTA and DPA n-6’ pattern [44]. Particularly, the ‘high EPA and DHA; low DTA and DPA n-6’ pattern, characterized by high concentrations of EPA and DHA and low levels of DTA and DPA, could be broadly classified as a ‘high marine FAs’ pattern. EPA, DHA, DTA and DPA, constituents of marine-derived FAs, are notably abundant in marine food sources, with fish being a primary example [45]. While little is known regarding the biologically connection between the two medium-chain saturated FAs (capric acid and lauric acid) and GLA and SDA, there are some hypotheses worth considering. For instance, Goel et al. suggested that capric acid might reduce PUFA biohydrogenation, leading to the accumulation of hydrogenation intermediates, particularly at high doses [46]. Furthermore, our observation of different groupings between samples showing ‘high capric acid and lauric acid’ pattern and those displaying the ‘low capric acid and lauric acid; high GLA and SDA’ pattern suggests the need for caution when interpreting the latter’s results.

Prior studies have provided evidence for the negative association of dietary fish intake with the prevalence of mental disorders, including depression and anxiety [46–48]. However, the findings on the relationship between individual marine FA concentration and depression have been inconsistent. For instance, the Three-City Study found that plasma EPA but not DHA was related to a decreased risk of depression [49], while another prospective study indicated an inverse association of depression with both EPA and DHA concentrations in blood [50]. In contrast, a secondary analysis of a krill oil supplementation trial and a cross-sectional study found no evidence for associations of DHA and EPA with depression [21, 51]. The inconsistency in these findings by examining individual FA effects may be attributed to simultaneous exposure to multiple FAs and their interactions. Using the PCA, our study offers a more comprehensive assessment of the association between FAs and depression, thereby furnishing theoretical substantiation for a multifaceted FAs strategy in depression prevention. We encourage forthcoming research to replicate our favorable findings using an a priori approach, such as reduced rank regression.

In the current study, the positive relationship between the ‘low capric acid and lauric acid; high GLA and SDA’ pattern score and the OR of depression was observed. Capric acid and lauric acid are median chain FAs that have been suggested to have a therapeutic effect on mood disorders [52, 53]. These two FAs provide ketone bodies such as acetate and β-hydroxy butyrate and protect cortical neurons against amyloid β-induced toxicity [54]. Additionally, rodent animal models showed that coconut oil that contains a high amount of lauric acid can prevent stress-induced depressive- and anxiety-like behaviors [55]. In contrast, GLA and SDA are intermediate metabolites of the DHA and EPA synthesis pathways, respectively, and were found in relatively high abundance within plant seed oils [56, 57]. Studies have indicated that dietary supplementations with GLA and SDA can reduce the pro-inflammatory states, an underlying etiological mechanism for depression [58, 59]. Nevertheless, the limited conversion rates of GLA and SDA have led to uncertainties regarding their protective efficacy [57]. Lemke et al. reported that the effects of SDA supplementation at 4.2 g a day were only comparable to those of 1 g/day EPA supplement, suggesting a relative inefficiency and marginal influence of SDA [60]. Consequently, the potency of elevated GLA and SDA concentrations may not be sufficient to obscure the effects of concurrent low capric acid and lauric acid levels, potentially elucidating our positive findings. To note, the results of the ‘low capric acid and lauric acid; high GLA and SDA’ pattern from sensitivity analyses were inconsistent with the preliminary results, warranting cautious interpretation of these findings.

In the current study, smokers and drinkers had a reduced OR of depression in participants with the highest tertiles of ‘high EPA and DHA; low DTA and DPA n-6’ pattern score. Some research suggested a positive association between alcohol consumption, smoking and the severity of depressive symptoms [61, 62]. Meanwhile, prior studies have reported that circulating n-3 long-chain PUFAs, including EPA and DHA, appeared lower in smokers and drinkers, which were not solely accounted for by dietary intake variations but rather by their bioavailability [63–65]. For example, Pawlosky et al. found in an intervention study that smoking decreased the conversion of linolenic acid to EPA [63]. Thus, smokers and drinkers within the highest tertiles of the ‘high EPA and DHA; low DTA and DPA n-6’ pattern score might possess an increased supply to sustain elevated levels of EPA and DHA, potentially resulting in a more pronounced impact on depression. There is also a notion that nicotine and ethanol work to deliver a “mood boost” by impacting the pleasure centers of the brain, thus potentially being a solution to relieving symptoms of depression [66, 67].

Our results also showed that the associations of the FA patterns of ‘high EPA and DHA; low DTA and DPA n-6’ and the ‘low capric acid and lauric acid; high GLA and SDA’ with depression were only significant for females. As essential components of lipids, the FAs’ in vivo actions are likely to be affected by the types and levels of hormones [68]. Recent studies have highlighted the effects of sex hormone administration upon the mRNA expression of key enzymes involved in the endogenous synthesis of longer-chain FAs [69]. Studies conducted in rat models demonstrated that females have significantly higher mRNA expression of Δ5 and Δ6 desaturases [70]. Concerning the age effect, it has been observed that depression in older adulthood differs from that in other age groups, especially in terms of comorbidities with other psychiatric disorders and varied responses to similar interventions [71]. In contrast to young adults, older adults’ depression is more closely related to cognitive impairments, cardio- and cerebrovascular diseases and dementia [72]. Our study observed the cumulative effect of high EPA and DHA exclusively among the elderly, underscoring the need for tailored FA treatment approaches when addressing depressive symptoms in older adults.

Compared to the individuals with obesity, non-obese individuals had a decreasing OR of depression in those with the highest tertile of ‘high EPA and DHA; low DTA and DPA n-6’ pattern score. Depression and obesity tend to co-occur within individuals and several longitudinal meta-analyses have affirmed a bidirectional relationship [73, 74]. The interconnection of these conditions involves adverse factors such as long sleep duration and stress, each potentially reinforcing the other [75]. Further, these factors, prevalent in individuals with obesity and depression, may influence the levels and effects of FAs [76, 77]. For instance, studies have found that stress can impact FAs, and FAs, in turn, are linked to sleep duration [78, 79]. Therefore, it is imperative to consider these variables, such as stress and daytime naps, as potential confounders in future studies examining the link between FAs and depression in individuals with obesity.

### Strengths and limitations

Our study is subject to several limitations. First, the cross-sectional design of the current study limits our ability to establish causality. Additionally, the potential unmeasured or residual confounding may affect our results. Adverse life events, for example, have been reported to be negatively associated with depression; however, this factor was not well measured in the current study, and therefore, its impact cannot be entirely ruled out. Second, while our study examined a more extensive range of FAs than previous studies, it only covered a subset of all possible FAs. A more comprehensive exploration of the association between FA patterns and depression remains an important topic for future research. Third, the relatively small number of patients with depression in subgroup analyses may have affected the accuracy of the observed association, so the results should be interpreted with caution. Fourthly, The PHQ-9, being a self-report tool, is considered less clinically discriminating than clinician-administered assessments, which is pertinent in predicting the effectiveness of omega-3 supplements for depression [17]. Fifth, the multiple comparisons conducted in the current study may elevate the risk of type 1 errors [80]. Lastly, the FA concentrations were measured only in serum samples obtained at a single time point. It is important to note that serum FAs may not fully capture the FA composition present in erythrocytes or phospholipid fractions and, therefore do not provide the same information. Nonetheless, it has been reported that red blood cell membrane FAs reflected long-term effect status, and moderate-to-strong correlations between FAs in the cell-free fraction of blood and red blood cell membrane have been observed [81].

## Conclusions

Our findings highlight the association between serum FA patterns and depression, with variations observed among subgroups across specific FA patterns. Prospective research, including cohort studies and basic experimental investigations, is needed to establish the association between novel FA patterns and depression and elucidate the underlying mechanism.

### Electronic supplementary material

Below is the link to the electronic supplementary material.

Supplementary Material 1

## Data Availability

The datasets collected and examined in this research can be found at [https://www.cdc.gov/nchs/nhanes/index.htm], the NHANES repository.

## Abbreviations

FAs: Fatty acids
NHANES: National Health and Nutrition Examination Survey
PUFA: Polyunsaturated fatty acids
MUFA: Monounsaturated fatty acids
SFA: Saturated fatty acids
TFA: Trans fatty acids
DHA: Docosahexaenoic acid
EPA: Eicosapentaenoic acid
ALA: Alpha-linolenic acid
SDA: Stearidonic acid
DHA: Docosahexaenoic acid
DPA: Docosapentaenoic acid
PCA: Principal component analysis
NCHS: National Center for Health Statistics
CDC: Centers for Disease Control and Prevention
PHQ-9: The Patient Health Questionnaire
LOD: The limit of detection
LCSFA: Long-chain saturated fatty acids
MCSFA: Median chain saturated fatty acids
DTA: Docosatetraenoic acid
GLA: Gamma-linolenic acid
LCFA: Long-chain fatty acids
DAG: Directed acyclic graph
BMI: Body mass index
