# Supplementary material for: Association of serum fatty acid pattern with depression in U.S. adults: analysis of NHANES 2011–2012
Source: Lipids Health Dis. 2024 Jun 8;23:177. doi: 10.1186/s12944-024-02142-9 (PMC11161977; doi:10.1186/s12944-024-02142-9)
Supplement: Supplementary file 1 — Supplementary Material 1 [file 12944_2024_2142_MOESM1_ESM.docx]

Table S1 The list of 30 subtypes of fatty acids included in the principal component analysis.

| Saturated Fatty Acids (SFA) | Monounsaturated Fatty Acids (MUFA) | Polyunsaturated Fatty Acids (PUFA) |
| --- | --- | --- |
| Capric acid (C10:0) | Myristoleic acid (14:1n-5) | Linoleic acid (18:2n-6) |
| Lauric acid (C12:0) | Palmitoleic acid (16:1n-7) | alpha-Linolenic acid (18:3n-3) |
| Myristic acid (14:0) | cis-Vaccenic acid (18:1n-7) | gamma-Linolenic acid (18:3n-6) |
| Pentadecanoic acid (C15:0) | Oleic acid (18:1n-9) | Stearidonic acid (C18:4n-3) |
| Palmitic acid (16:0) | Eicosenoic acid (20:1n-9) | Eicosadienoic acid (20:2n-6) |
| Margaric acid (C17:0) | Nervonic acid (24:1n-9) | homo-gamma-Linolenic acid (20:3n-6) |
| Stearic acid (18:0) |  | Eicosatrienoic acid (C20:3n-9) |
| Arachidic acid (20:0) |  | Arachidonic acid (20:4n-6) |
| Docosanoic acid (22:0) |  | Eicosapentaenoic acid (20:5n-3) |
| Tricosanoic acid (C23:0) |  | Docosatetraenoic acid (22:4n-6) |
| Lignoceric acid (24:0) |  | Docosapentaenoic acid (22:5n-3) |
|  |  | Docosapentaenoic acid (22:5n-6) |
|  |  | Docosahexaenoic acid (22:6n-3) |

Table S2 Baseline characteristics of study participants by tertiles of serum fatty acids pattern scores.

| Characteristics | ’High LCSFA‘ pattern | | | *p*-value^1^ | ’Low MCSFA and Myristoleic acid‘ pattern | | | *p*-value^1^ | ’High EPA and DHA; Low DTA and DPA‘ pattern | | | *p*-value^1^ | ’Low Capric acid and Lauric acid; High GLA and SDA‘ pattern | | | *p*-value^1^ |
| --- | --- | --- | --- | --- | --- | --- | --- | --- | --- | --- | --- | --- | --- | --- | --- | --- |
|  | Tertile 1 | Tertile 2 | Tertile 3 |  | Tertile 1 | Tertile 2 | Tertile 3 |  | Tertile 1 | Tertile 2 | Tertile 3 |  | Tertile 1 | Tertile 2 | Tertile 3 |  |
| Age (years), Mean ± SD | 47.10 ± 18.80 | 43.04 ± 15.95 | 46.23 ± 17.26 | 0.04 | 44.83 ± 15.70 | 45.75 ± 18.17 | 46.36 ± 17.63 | 0.81 | 41.94 ± 16.07 | 44.35 ± 16.68 | 50.87 ± 17.76 | 0.01 | 39.91 ± 16.31 | 44.98 ± 16.84 | 52.69 ± 16.12 | <0.01 |
| BMI (kg/m2), Mean ± SD | 29.53 ± 7.29 | 29.42 ± 7.34 | 29.05 ± 6.50 | 0.94 | 30.77 ± 6.00 | 29.35 ± 6.44 | 27.57 ± 7.37 | <0.01 | 29.19 ± 7.84 | 29.81 ± 6.12 | 28.49 ± 6.24 | 0.36 | 27.53 ± 7.07 | 30.12 ± 6.73 | 30.03 ± 6.12 | 0.02 |
| Total energy (kcal), Mean ± SD | 2,020.36 ± 802.32 | 2,175.91 ± 1,035.24 | 2,125.70 ± 747.78 | 0.14 | 2,265.96 ± 931.89 | 2,121.97 ± 815.30 | 1,994.87 ± 680.10 | 0.07 | 2,142.21 ± 833.28 | 2,159.28 ± 804.88 | 2,069.03 ± 818.10 | 0.24 | 2,170.02 ± 902.09 | 2,128.25 ± 777.47 | 2,068.00 ± 761.75 | 0.73 |
| Sex, n (%) |  |  |  | 0.48 |  |  |  | 0.04 |  |  |  | 0.04 |  |  |  | 0.11 |
| Male | 123 (43.00) | 128 (49.69) | 119 (48.18) |  | 149 (55.06) | 133 (51.46) | 88 (37.78) |  | 133 (54.85) | 128 (49.61) | 109 (39.06) |  | 117 (40.87) | 117 (49.27) | 136 (54.11) |  |
| Female | 140 (57.00) | 136 (50.31) | 145 (51.82) |  | 114 (44.94) | 131 (48.54) | 176 (62.22) |  | 130 (45.15) | 137 (50.39) | 154 (60.94) |  | 146 (59.13) | 148 (50.73) | 127 (45.89) |  |
| Physical activity, n (%) |  |  |  | 0.46 |  |  |  | 0.02 |  |  |  | 0.15 |  |  |  | 0.66 |
| Not reported | 143 (52.24) | 136 (51.06) | 121 (42.82) |  | 135 (42.30) | 141 (53.79) | 124 (40.66) |  | 134 (49.46) | 140 (48.28) | 126 (38.91) |  | 124 (44.96) | 125 (43.41) | 151 (48.64) |  |
| <500 Met-min/week | 34 (13.50) | 37 (13.46) | 41 (17.58) |  | 41 (23.67) | 37 (14.73) | 35 (11.00) |  | 35 (15.51) | 40 (18.42) | 37 (14.36) |  | 39 (13.42) | 46 (19.47) | 28 (16.09) |  |
| 500-1000 Met-min/week | 32 (13.40) | 28 (10.69) | 35 (13.05) |  | 33 (13.57) | 21 (7.15) | 41 (17.06) |  | 29 (13.27) | 28 (7.83) | 38 (17.35) |  | 33 (13.12) | 36 (14.03) | 26 (10.57) |  |
| >1000 Met-min/week | 54 (20.86) | 63 (24.79) | 67 (26.54) |  | 54 (20.46) | 65 (24.33) | 64 (31.28) |  | 65 (21.76) | 57 (25.47) | 62 (29.38) |  | 67 (28.50) | 58 (23.09) | 58 (24.71) |  |
| Race/ethnicity, n (%) |  |  |  | <0.01 |  |  |  | <0.01 |  |  |  | <0.01 |  |  |  | 0.55 |
| Mexican American | 48 (15.39) | 91 (37.17) | 31 (6.06) |  | 70 (16.19) | 60 (14.44) | 40 (10.34) |  | 62 (16.61) | 62 (14.18) | 46 (9.90) |  | 64 (16.19) | 50 (11.49) | 56 (12.89) |  |
| Non-Hispanic whites | 25 (8.57) | 81 (30.51) | 225 (92.55) |  | 124 (73.03) | 108 (69.39) | 98 (67.35) |  | 114 (65.85) | 113 (71.76) | 104 (71.88) |  | 105 (67.32) | 114 (71.20) | 111 (71.18) |  |
| Non-Hispanic black | 121 (51.98) | 66 (23.40) | 3 (0.35) |  | 35 (5.82) | 60 (10.81) | 95 (16.48) |  | 74 (14.97) | 63 (10.48) | 52 (7.93) |  | 58 (9.97) | 70 (12.89) | 62 (10.76) |  |
| Others | 69 (24.06) | 26 (8.92) | 5 (1.04) |  | 34 (4.97) | 36 (5.36) | 31 (5.82) |  | 13 (2.58) | 27 (3.58) | 61 (10.29) |  | 36 (6.52) | 31 (4.42) | 34 (5.18) |  |
| Education, n (%) |  |  |  | <0.01 |  |  |  | 0.33 |  |  |  | 0.01 |  |  |  | 0.76 |
| Less than 9th grade or 9–11th grade | 59 (21.73) | 74 (30.39) | 32 (11.22) |  | 58 (13.81) | 56 (18.18) | 51 (16.90) |  | 66 (24.60) | 42 (11.35) | 57 (14.11) |  | 46 (15.34) | 53 (17.79) | 66 (15.92) |  |
| High school graduate | 50 (21.66) | 62 (22.86) | 53 (17.70) |  | 61 (20.71) | 64 (22.91) | 40 (14.26) |  | 68 (23.12) | 54 (21.18) | 43 (13.38) |  | 40 (15.75) | 60 (20.20) | 65 (21.81) |  |
| Some college or AA degree | 68 (29.27) | 80 (32.17) | 89 (33.93) |  | 87 (39.96) | 72 (27.52) | 77 (32.04) |  | 73 (30.44) | 95 (36.67) | 68 (31.30) |  | 80 (36.55) | 78 (30.35) | 78 (32.13) |  |
| College graduate or above | 67 (27.35) | 39 (14.58) | 84 (37.14) |  | 48 (25.51) | 58 (31.40) | 84 (36.79) |  | 37 (21.83) | 63 (30.80) | 90 (41.20) |  | 71 (32.36) | 66 (31.67) | 53 (30.13) |  |
| Annual family income, n (%) |  |  |  | <0.01 |  |  |  | 0.88 |  |  |  | <0.01 |  |  |  | 0.20 |
| Under $20,000 | 82 (32.95) | 97 (37.97) | 51 (13.22) |  | 87 (21.49) | 77 (19.99) | 66 (20.08) |  | 98 (28.91) | 77 (21.40) | 56 (11.11) |  | 73 (18.62) | 80 (24.97) | 77 (17.94) |  |
| $20,000 and over | 167 (67.05) | 154 (62.03) | 207 (86.78) |  | 170 (78.51) | 173 (80.01) | 185 (79.92) |  | 157 (71.09) | 175 (78.60) | 195 (88.89) |  | 180 (81.38) | 172 (75.03) | 176 (82.06) |  |
| Marital status, n (%) |  |  |  | 0.03 |  |  |  | 0.12 |  |  |  | 0.14 |  |  |  | 0.14 |
| Married or living with partner | 130 (51.27) | 109 (44.25) | 158 (63.08) |  | 138 (58.76) | 128 (51.46) | 131 (63.26) |  | 103 (50.01) | 150 (61.56) | 143 (61.12) |  | 129 (59.09) | 125 (56.21) | 143 (58.31) |  |
| Widowed/divorced/separated | 63 (23.13) | 81 (30.34) | 47 (16.53) |  | 71 (23.72) | 55 (18.18) | 64 (18.66) |  | 79 (23.58) | 48 (15.27) | 64 (22.25) |  | 40 (15.00) | 70 (20.20) | 80 (25.35) |  |
| Never married | 51 (25.60) | 65 (25.41) | 53 (20.40) |  | 45 (17.52) | 67 (30.36) | 57 (18.08) |  | 62 (26.41) | 56 (23.17) | 51 (16.64) |  | 68 (25.90) | 62 (23.59) | 39 (16.35) |  |
| Smoking, n (%) |  |  |  | 0.91 |  |  |  | 0.02 |  |  |  | 0.01 |  |  |  | 0.06 |
| Yes | 108 (42.32) | 109 (42.31) | 119 (43.52) |  | 144 (53.58) | 106 (43.52) | 85 (33.18) |  | 130 (51.99) | 108 (46.10) | 97 (31.32) |  | 85 (34.78) | 118 (44.73) | 132 (50.23) |  |
| No | 135 (57.68) | 146 (57.69) | 139 (56.48) |  | 110 (46.42) | 143 (56.48) | 167 (66.82) |  | 114 (48.01) | 145 (53.90) | 161 (68.68) |  | 151 (65.22) | 139 (55.27) | 130 (49.77) |  |
| Drinking alcohol, n (%) |  |  |  | <0.01 |  |  |  | 0.26 |  |  |  | 0.94 |  |  |  | 0.53 |
| Yes | 174 (66.69) | 185 (71.81) | 226 (88.04) |  | 203 (86.06) | 189 (80.39) | 192 (80.34) |  | 203 (81.57) | 192 (82.10) | 189 (82.79) |  | 192 (80.49) | 201 (82.19) | 191 (83.98) |  |
| No | 88 (33.31) | 79 (28.19) | 38 (11.96) |  | 60 (13.94) | 74 (19.61) | 72 (19.66) |  | 59 (18.43) | 73 (17.90) | 74 (17.21) |  | 71 (19.51) | 64 (17.81) | 71 (16.02) |  |

^1^: *P* value was obtained from Chi-square test or Fisher’s exact when appropriate.

Table S3 Linear regression analyses of the association of serum fatty acids pattern score with PHQ score in sensitivity analyses.

| Variables | Tertile 1 | Tertile 2 | Tertile 3 | *P*-trend |
| --- | --- | --- | --- | --- |
| 'high LCSFA and LCFA' pattern |  |  |  |  |
| Model 1 | Ref. | 0.61 (-0.37, 1.59) | -0.45 (-1.54, 0.64) | 0.16 |
| Model 2 | Ref. | 0.66 (-0.30, 1.62) | -0.41 (-1.47, 0.65) | 0.17 |
| Model 3 | Ref. | 0.44 (-0.52, 1.41) | -0.01 (-1.15, 1.13) | 0.71 |
| Model 4 | Ref. | 0.57 (-0.47, 1.61) | 0.20 (-1.03, 1.43) | 0.95 |
| 'low MCSFA and myristoleic acid' pattern |  |  |  |  |
| Model 1 | Ref. | -0.77 (-1.99, 0.45) | 0.45 (-1.17, 2.07) | 0.23 |
| Model 2 | Ref. | -0.80 (-1.94, 0.33) | 0.05 (-1.34, 1.44) | 0.25 |
| Model 3 | Ref. | -0.76 (-2.20, 0.67) | 0.45 (-1.17, 2.07) | 0.06 |
| Model 4 | Ref. | -0.56 (-2.19, 1.07) | 0.83 (-0.72, 2.38) | 0.24 |
| 'high EPA and DHA; low DTA and DPA' pattern |  |  |  |  |
| Model 1 | Ref. | -0.14 (-1.16, 0.87) | -1.19 (-2.18, -0.21)^*^ | 0.03 |
| Model 2 | Ref. | -0.20 (-1.14, 0.73) | -1.38 (-2.48, -0.28)^*^ | 0.03 |
| Model 3 | Ref. | -0.22 (-1.19, 0.74) | -1.23 (-2.26, -0.21)^*^ | 0.04 |
| Model 4 | Ref. | -0.05 (-1.17, 1.07) | -0.84 (-1.63, -0.05)^*^ | 0.06 |
| 'low capric acid and lauric acid; high GLA and SDA' pattern |  |  |  |  |
| Model 1 | Ref. | 1.06 (0.18, 1.95) ^*^ | 0.35 (-0.13, 0.82) | 0.14 |
| Model 2 | Ref. | 1.18 (0.25, 2.11) ^*^ | 0.57 (0.02, 1.11) ^*^ | 0.04 |
| Model 3 | Ref. | 0.99 (-0.05, 2.03) | 0.38 (-0.35, 1.10) | 0.35 |
| Model 4 | Ref. | 0.96 (0.03, 1.88) ^*^ | 0.36 (-0.39, 1.10) | 0.36 |

Ref: reference; PHQ: Patient Health Questionnaire. ^*^: *P*<0.05.

Model 1: Crude model.

Model 2: adjusted for age and sex.

Model 3: adjusted for model 2 plus race/ethnicity, education, marital status, annual family income, body mass index, alcohol status and smoking status.

Model 4: adjusted for model 3 plus physical activity and total energy intake.

Table S4 Odds ratios and 95% confidential intervals for depression (PHQ>9) according to the tertiles of serum fatty acid pattern score using concentration as the primary exposure

| Variables | Tertile 1 | Tertile 2 | Tertile 3 | *P*-trend |
| --- | --- | --- | --- | --- |
| 'high LCSFA and LCFA' pattern |  |  |  |  |
| Model 1 | Ref. | 1.45 (0.70, 3.00) | 1.11 (0.56, 2.18) | 0.85 |
| Model 2 | Ref. | 1.45 (0.70, 2.99) | 1.15 (0.58, 2.29) | 0.98 |
| Model 3 | Ref. | 1.16 (0.54, 2.49) | 1.38 (0.63, 3.01) | 0.43 |
| Model 4 | Ref. | 1.12 (0.48, 2.59) | 1.46 (0.62, 3.47) | 0.36 |
| ‘low MCSFA and myristoleic acid' pattern |  |  |  |  |
| Model 1 | Ref. | 0.81 (0.44, 1.50) | 0.77 (0.37, 1.64) | 0.58 |
| Model 2 | Ref. | 0.77 (0.40, 1.48) | 0.72 (0.32, 1.61) | 0.43 |
| Model 3 | Ref. | 1.01 (0.53, 1.92) | 0.85 (0.44, 1.65) | 0.62 |
| Model 4 | Ref. | 1.01 (0.53, 1.90) | 0.86 (0.44, 1.71) | 0.69 |
| 'high EPA and DHA; low DTA and DPA' pattern |  |  |  |  |
| Model 1 | Ref. | 2.04 (0.97, 4.29) | 3.70 (1.68, 8.13)^*^ | <0.01 |
| Model 2 | Ref. | 2.19 (0.91, 5.24) | 3.99 (1.70, 9.36)^*^ | <0.01 |
| Model 3 | Ref. | 1.81 (0.67, 4.86) | 3.46 (1.44, 8.33)^*^ | 0.03 |
| Model 4 | Ref. | 1.75 (0.64, 4.81) | 3.35 (1.42, 7.93)^*^ | 0.11 |
| 'low capric acid and lauric acid; high GLA and SDA' pattern |  |  |  |  |
| Model 1 | Ref. | 1.01 (0.52, 1.96) | 0.78 (0.37, 1.65) | 0.50 |
| Model 2 | Ref. | 0.92 (0.51, 1.66) | 0.67 (0.31, 1.45) | 0.32 |
| Model 3 | Ref. | 1.09 (0.56, 2.14) | 0.68 (0.30, 1.54) | 0.40 |
| Model 4 | Ref. | 1.10 (0.56, 2.18) | 0.68 (0.30, 1.53) | 0.66 |

Ref: reference; PHQ: Patient Health Questionnaire. ^*^: *P*<0.05.

Model 1: Crude model.

Model 2: adjusted for age and sex.

Model 3: adjusted for model 2 plus race/ethnicity, education, marital status, annual family income, body mass index, alcohol status and smoking status.

Model 4: adjusted for model 3 plus physical activity and total energy intake.

*P*-trend was obtained using the tertiles as a continuous variable.

Supplementary Figure S1. Scree plot of principal component analysis. (a) bar plot of percentage of explained variance for each component. (b) bar plot of eigenvalues for each component.





Supplementary Figure S2. Directed acyclic graph (DAG) for the association between serum fatty acid pattern and depression.

Supplementary Figure S3. Scree plot of principal component analysis using concentration as the primary exposure. (a) bar plot of percentage of explained variance for each component. (b) bar plot of eigenvalues for each component.

Supplementary Figure S4. Factor loading matrix of fatty acids for the major serum fatty acids pattern using concentration as the primary exposure. LCSFA: long-chain saturated fatty acids, MCSFA: medium-chain saturated fatty acids, LCFA: long-chain fatty acids, EPA: eicosapentaenoic acid, DHA: docosahexaenoic acid, DPA: docosapentaenoic acid, DTA: docosatetraenoic acid, GLA: gamma-linolenic acid, SDA: stearidonic acid.
